# Mapping, Measuring, and Analyzing the Process of Skin-to-Skin Contact and Early Breastfeeding in the First Hour After Birth

**DOI:** 10.1089/bfm.2018.0048

**Published:** 2018-09-12

**Authors:** Karin Cadwell, Kajsa Brimdyr, Raylene Phillips

**Affiliations:** ^1^ Maternal Child Health, Healthy Children Project, Inc., East Sandwich, Massachusetts.; ^2^ Loma Linda University Medical Center, Loma Linda, California.

**Keywords:** skin-to-skin, birth, breastfeeding initiation, Baby-Friendly hospital

## Abstract

***Background:*** Although the benefits of immediate, continuous, uninterrupted skin-to-skin contact (SSC) and early breastfeeding have been widely researched and confirmed, the challenge remains to improve the consistency of this practice. Fewer than half of newborns worldwide are breastfed in the first hour.

***Design:*** Cross-sectional descriptive study utilizing iterative review and analysis of video ethnography as well as data extracted from patient records.

***Sample and Setting:*** Eighty-four medically uncomplicated mothers and full-term newborns were observed during the first hour after birth at a Baby-Friendly designated hospital in the United States.

***Findings:*** Process mapping using an algorithm which included Robson criteria indicated that although included mothers were expected to give birth vaginally and had no medical concerns that would preclude eligibility for SSC in the first hour after birth, 31 of 84 newborns (37%) did not receive immediate SSC after vaginal birth as planned and only 23 (27.4%) self-attached and suckled.

***Conclusion:*** Process mapping of optimal skin-to-skin practice in the first hour after birth using the algorithm, HCP-S2S-IA, produced an accurate and useful measurement, illuminating how work is conducted and providing patterns for analysis and opportunities for improvement with targeted interventions.

## Introduction

The WHO Baby-Friendly Clinical Guidance released in 2018 continues to clarify the original Step 4 of the 10 Steps to Successful Breastfeeding by stating that birthing facilities should “facilitate immediate and uninterrupted skin-to-skin contact and support mothers to initiate breastfeeding as soon as possible after birth,” (p.17), adding that it “should be uninterrupted for at least 60 minutes.” (p.25)^1^ Placing the newly born infant skin-to-skin, ventral surface to ventral surface, immediately after birth and allowing the dyad to remain together continuously and without interruption until the completion of the first breastfeed is specified in the Baby-Friendly USA^2^ Step 4 standard “unless there are documented medically justifiable reasons for delayed contact or interruption.” (p.14) The most recent Cochrane review of the practice of early skin-to-skin contact (SSC) for mothers and their healthy newborn infants^3^ concludes, “since we found no evidence of harm in any included studies, we conclude the evidence supports that early SSC should be normal practice for healthy newborns, including those born by cesarean and babies born early at 35 weeks or more.”(p.3)

SSC benefits the infant physiologically in the hours and days after birth by decreasing the stress of being born^4,5^ promoting more optimal thermal regulation,^6^ decreasing crying,^7,8^ improving cardiopulmonary dynamics,^5^ and resulting in more optimal blood glucose levels.^3^ Research examining the early benefits of SSC for the mother include a shorter third stage of labor,^9^ along with immediate contraction of the uterus, more complete placental separation,^10^ and reduction in blood loss.^10,11^ In the longer term, SSC has been demonstrated to improve the mother's breastfeeding self-efficacy,^12^ lead to more optimal suckling as assessed in the second week,^13^ and to increase the probability of exclusive breastfeeding.^14–18^

When SSC begins immediately after birth and the baby remains in SSC continuously and without interruption, he or she seeks the breast, progresses in a predictable fashion through Widström's 9 Stages,^19^ (Table 1) self-attaches and begins to suckle (eighth stage), usually within the first hour or so. Delayed breastfeeding initiation (past the first hour after birth) increases the risk of neonatal mortality. A recent systematic review and meta-analysis of studies from both high and low income countries found that infants who initiated breastfeeding between 2 and 23 hours after birth compared to those who initiated breastfeeding within the first hour after birth, had a 33% increased risk of neonatal mortality.^20^

**Table 1.** Widström's 9 Stages

Babies progress through nine observable, instinctive stages during the first hour after birth when in immediate, continuous, and uninterrupted skin-to-skin contact with the mother. Stage 8 is suckling, the first experience of breastfeeding.
1. The *birth cry* is a distinct and specific cry as the baby's lungs expand for the first time.
2. *Relaxation* is a time immediately after the birth cry ends, when the baby becomes still and has no visible movements.
3. *Awakening* begins as the baby opens the eyes for the first time, blinks, has small mouth movements, and limited hand and shoulder motions.
4. *Activity* involves larger body movements, including whole arm motions, specific finger movements, shoulder motion, head lifting, and stable open eyes.
5. *Rest* could happen at any point during the first hour, interspersed between stages or as a transition between stages.
6. *Crawling* involves the baby moving purposely toward the breast and nipple. It could be accomplished through sliding, leaping, bobbing, or pushing.
7. *Familiarization* is a stage at the mother's nipple where the baby licks, tastes, touches, and moves around the nipple and areola area.
8. *Suckling* involves the baby self-attaching to the nipple and initiating breastfeeding.
9. *Sleeping* is an involuntary activity of the baby around 1.5 to 2 hours after birth.

Adapted from Widström et al.^19^

Although the benefits of immediate, continuous, uninterrupted SSC and early breastfeeding have been widely researched and confirmed, fewer than half of newborns worldwide are breastfed in the first hour. According to UNICEF,^21^ “This leaves 77 million newborns waiting too long for this first critical contact with their mother outside of the womb.” (p.8) Inconsistency in the practice of SSC has been highlighted in the most recent Cochrane review of early SSC for mothers and their healthy newborn infants.^3^ Just 47% of the 38 trials eligible for inclusion in the analysis reported that SSC began “early” or “immediately.” Sixty-six studies were assessed and excluded from the review^3^ primarily because “… the investigators did not state that the infants in the intervention group received immediate or early skin-to-skin contact.” (p.8) There is also inconsistency in duration of SSC with published ranges of time from 15 minutes^22,23^ to more than 30 hours.^3^

The purpose of this study was to analyze the process of uninterrupted SSC between healthy newborns and their mothers immediately after planned vaginal birth in a hospital that had implemented this practice change for both vaginal and cesarean births.

## Materials and Methods

### Design

This research design was a cross-sectional descriptive study utilizing video ethnographic iterative review and analysis as well as data extracted from patient records.

### Sample and setting

The study was conducted at Loma Linda University Medical Center (LLUMC), a large teaching hospital with ∼2,500 births per year. The hospital was designated Baby-Friendly^®^ in 2009, and redesignated in 2014, and is located in California, a state with one of the highest breastfeeding rates in the United States.^24^

Hospitals designated “Baby-Friendly” by the U.S. authority, Baby-Friendly USA, are expected to implement the routine practice of helping new mothers breastfeed within the first hour after birth. This is interpreted as immediate, continuous, uninterrupted SSC for all healthy newborns for at least 1 hour after both vaginal and cesarean births whether or not the mother plans to breastfeed. If the mother plans to breastfeed, SSC should continue until the completion of the first breastfeed. The staff at the study hospital had received a 5-day in-service training on the importance of, and application of, skin-to-skin using the PRECESS method.^15^ This observational study, approved by the hospital's Institutional Review Board, included mothers who were planning a vaginal birth. The protocol included video recording stable babies and mothers in SSC during the first hour after birth and documenting the medications mothers had received during labor.

Over the course of 4 weeks, 1 week each in May, July, August, and December 2013, informed consent was obtained from clinically uncomplicated nulliparas and multiparas planning a vaginal delivery. Mothers were approached upon arrival to the labor and delivery ward. Informed consent materials were available in English and Spanish, with translation services available for Spanish-speaking participants. The study's inclusion criteria included women who were ≥18 years of age, low risk (as determined by the admitting doctor), English or Spanish speaking as their primary language, and who were estimated to be between 37 and 42 weeks gestation. Infants were eligible if they were full-term gestation, stable, and had no known abnormalities. To safeguard protected health information, names and identifying information were removed from data collection material, which were assigned a linked code. This unique code was also recorded on the video of the baby taken during the first hour after birth.

Ninety-six mothers consented to participate in the study. Five had been scheduled for repeat cesareans, and therefore did not meet the inclusion criteria and were considered mistakenly consented. Other participants removed from study analysis included one mother with a previously undisclosed physical condition that would preclude breastfeeding and one who was recategorized as at less than 37 weeks gestation after being consented. One mother was removed after the baby was born with an unexpected cleft. Of these 88, 4 did not include para status in the medical record, resulting in 84 dyads in the final group who could be categorized by Robson criteria. Standard procedures were followed for all study participants, with the exception of the addition of the video recording of the baby for the first hour after birth while skin-to-skin with the mother.

### Data collection procedures

Immediately after the birth, video recording of the mother and baby commenced. Hospital protocol stated that the baby would remain in SSC with the semireclined mother for at least the first hour after birth unless there was a medical reason to discontinue. The baby was allowed to move, uninterrupted, through Widström's 9 Stages (Table 1). The study protocol included the provision that if the baby was removed by the nurse or by NICU staff for more than 10 minutes, the video recording would be stopped. The dyad was then documented as “removed for medical reasons.” Demographics and medications received during labor were collected from the Electronic Medical Record System. Labor and delivery medications followed standard hospital protocols. Providers and staff prescribe, administer, and record each participant's medications in an Electronic Medical Record.

Robson criteria,^25^ a well-established method of monitoring and auditing, is used worldwide to standardize comparison methods between hospitals, systems, and countries. Births are categorized as 1 of 10 groups based on five parameters: obstetric history (parity and previous cesarean section), onset of labor (spontaneous, induced, or cesarean section before onset of labor), fetal presentation or lie (cephalic, breech, or transverse), number of neonates, and gestational age (preterm or term). Appropriate data were accessed from the patient record for each mother to determine the Robson criteria and allow for analysis of the variance in experience of nulliparas and multiparas, of induced mothers, or of mothers who had a vaginal birth after cesarean (VBAC).

The process measurement tool, the *Healthy Children Project Skin-to-Skin Implementation Algorithm*, (HCP-S2S-IA) was used to categorize the prebirth Robson criteria as well as operationalize the collection of specific measurable elements of the SSC practice. The tool has been shown to be relevant and has been applied in hospital settings for both vaginal and cesarean births in Australia and Japan. Analysis of collected data has been shown to provide insight into where dyads remain on, or leave, the pathway of best practice.^26^

### Video analysis

Iterative analysis of the video recording of each dyad was performed by two research assistants who had been blinded to patient records. The assistants had been trained to identify each of Widström's 9 Stages of newborn behavior. The training involved viewing a professional video^27^ that defined and illustrated each stage and then attending a 2-hour workshop about Widström's 9 Stages. The two reviewers separately and independently coded all of the video recordings using MAXQDA 11.0.2, 2013 for the nine stages. This required an iterative process of repeatedly watching the videos to fine tune the start and end time of each stage, and the elements of behavior of the newborn during that time. Disagreements were resolved through conversation with the trainer.

## Results

**FIG. 1.**
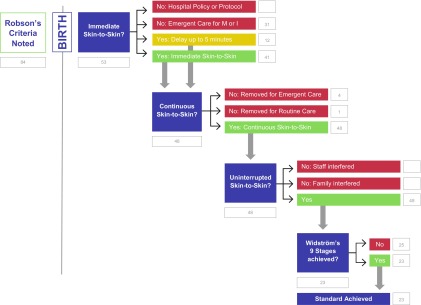
Healthy children project's skin‐to‐skin implementation algorithm with LLUMC data. LLUMC, Loma Linda University Medical Center.

The data in the Algorithm extracted from the video and patient record are displayed in Figure 1. This process map, HCP-S2S-IA, an algorithm, is color coded, with green indicating the pathway of best practice, yellow indicating practices for review, and red indicating practices that preclude achievement of immediate, uninterrupted, continuous skin-to-skin in the first hour. Robson criteria for each mother were determined from her hospital record and is displayed in Table 2 and sorted according to the data extracted from the algorithm in Table 3.

**Table 2.** Canadian Modified Robson Criteria

*Group*	*Description*
1	Nullipara, singleton, cephalic, ≥37 weeks, spontaneous labor
2	Nullipara, singleton, cephalic, ≥37 weeks
	(A) Induced
	(B) Cesarean section before labor
3	Multipara, singleton, cephalic, ≥37 weeks, spontaneous labor
4	Multipara, singleton, cephalic, ≥37 weeks
	(A) Induced
	(B) Cesarean section before labor
5	Previous cesarean section, singleton, cephalic, ≥37 weeks
	(A) Spontaneous labor
	(B) Induced
	(C) Cesarean section before labor
6	All nulliparous breeches
	(A) Spontaneous labor
	(B) Induced
	(C) Cesarean section before labor
7	All multiparous breeches (including previous cesarean section)
	(A) Spontaneous labor
	(B) Induced
	(C) Cesarean section before labor
8	All multiple pregnancies (including previous cesarean section)
	(A) Spontaneous labor
	(B) Induced
	(C) Cesarean section before labor
9	All abnormal lies (including previous cesarean section but excluding breech)
	(A) Spontaneous labor
	(B) Induced
	(C) Cesarean section before labor
10	All singleton cephalic, ≤36 weeks (including previous cesarean section)
	(A) Spontaneous labor
	(B) Induced
	(C) Cesarean section before labor

Adapted from Farine and Shepherd, 2012.^28^

**Table 3.** Tabulation of the Distribution of Study Participants According to Robson Criteria Against Skin-to-Skin Contact Progress Indicators

*Group*	*Pre-birth*	*Immediate hospital policy at birth*	*Immediate emergent care after birth*	*Immediate delayed <5 at birth*	*Immediate skin-to-skin*	*Continuous removed for emergent care*	*Continuous removed for routine care*	*Continuous*	*Uninterrupted staff interfered*	*Uninterrupted family interfered*	*Uninterrupted*	*Did not achieve 9 stages*	*Met standard*
1	23	0	6	2	15	2	0	15	0	0	15	8	7
2	A. 16	A. 0	A. 8	A. 4	A. 4	A. 1	A. 1	A. 6	A. 0	A. 0	A. 6	A. 6	A. 0
3	19	0	5	4	10	0	0	14	0	0	14	4	10
4	A. 15	A. 0	A. 5	A. 1	A. 9	A. 1	A.	A. 9	A. 0	A. 0	A. 9	A. 4	A. 5
5	A. 7	A. 0	A. 4	A. 0	A. 3	A. 0	A. 0	A. 3	A. 0	A. 0	A. 3	A. 2	A. 1
	B. 3	B. 0	B. 2	B. 1	B. 0	B. 0	B. 0	B. 1	B. 0	B. 0	B. 1	B. 1	
6	0												
7	0												
8	A. 1	A. 0	A. 1	A. 0	A. 0	A. 0	A. 0	A. 0	A. 0	A. 0	A. 0	A. 0	A. 0
9	0												
10	0												
Para unrecorded	(4)		(2)									(1)	(1)
	84	0	31	12	41	4	1	48	0	0	48	25	23

The number of women in each category of Robson criteria prebirth according to the patient record is indicated in the blue column. The yellow, green, and red columns match the colors of the pathways of the algorithm, HCP-S2S-IA. Green indicates best practice, yellow indicates a practice that should be reviewed, and red indicates dyads who left the best practice pathway. The dyads with missing data are recorded as “Para unrecorded.” These incomplete dyads are not included in the count, and are not included in the Algorithm.

### Prebirth Robson criteria

According to Robson criteria for this Algorithm (Table 3), 23 (27%) of the 84 participating women were first-time mothers with spontaneous labor (Robson criteria 1) and 16 (19.1%) were first-time mothers who were induced (Robson criteria 2). Nineteen (22.6%) were multiparas in spontaneous labor (Robson criteria 3), and 15 (18%) of the multiparas were induced (Robson criteria 4A). Ten (11.9%) of the mothers were categorized as Robson criteria 5, as they were laboring to have a VBAC, with 7 (8.3%) in spontaneous labor (5A), and 3 (3.6%) induced (5B). There was one mother who met the criteria for Robson 8, as she was anticipating a twin birth.

### Immediate SSC

The first criterion for optimal SSC practice is SSC immediately after birth. Immediate SSC was not observed in 10 (11.9%) of the dyads in this study due to emergent cesarean birth. Reasons for these emergent cesareans included fetal distress (three), nonreassuring fetal heart tracing (two), failed VBAC (one, whose newborn also had fetal distress and then respiratory failure), and failed inductions (four, whose newborns also had [one] transient tachypnea, [one] respiratory failure, [one] primary apnea of newborn, and [one] fetal heart murmur). Twenty-one (25%) did not get immediate SSC due to emergent care required for either the mother or the newborn after vaginal birth. Reasons for this included respiratory failure (five newborns), chorioamnionitis (four dyads), and postpartum hemorrhage (four mothers). Two newborns had fetal heart murmurs, one of which also had a mother who hemorrhaged. One of the mothers who hemorrhaged also had a uterine rupture and her newborn had respiratory failure. One newborn who was “jittery” went to the NICU. Two mothers refused their babies after the birth, and three had meconium-stained amniotic fluid so were taken directly to the exam table after birth before being returned to mother for SSC. Twelve (14.2%) of the dyads who did not receive SSC immediately after vaginal birth because of initial medical concerns did begin SSC within 5 minutes of birth. Fifty-three dyads (63.1%) met the standard by receiving immediate SSC after planned vaginal birth (either immediately after birth or within 5 minutes) (Fig. 1).

Of the 23 nulliparas who began labor spontaneously (Robson criteria 1), 1 (4.3%) was not observed to have immediate SSC due to emergent cesarean birth, and 5 (21.7%) did not get immediate SSC because of the need for emergent care for the mother or baby after vaginal birth. Of the 16 induced nulliparas (Robson criteria 2A), 4 (25%) were not observed to have SSC due to emergent cesarean birth, and 4 (25%) did not get immediate SSC due to the need for emergent care. Three (8.8%) of the 34 multiparas (Robson criteria 3 and 4A) were not observed to have SSC due to emergent cesarean birth (1 spontaneous labor and 2 induced), and 7 (21.2%) did not get immediate SSC because they required emergent care immediately after birth (4 spontaneous labor, 3 induced). Of the 10 VBAC dyads (Robson criteria 5A and B), 2 (20%) had emergent cesarean birth (1 spontaneous labor and 1 induced) and 4 (40%) required emergent care (3 spontaneous labor and 1 induced). The one set of twins (Robson criteria 8) required emergent care and did not experience immediate SSC.

### Continuous SSC

The second criterion of optimal SSC practice is continuous SSC. The process map Algorithm illustrates that 5 (9.4%) of the 53 dyads who began SSC immediately after birth were removed (4 for emergent care and 1 for routine care) and 48 (90.6%) continued on the best practice pathway. Of the five removed dyads, four were nulliparas (two in spontaneous labor [Robson 1A], two induced [Robson 2A], and one was an induced multipara [Robson 4A]). One induced nullipara (Robson 2A) was removed for the routine care of weighing and measuring the newborn. The newborns removed for emergent care were two spontaneous nulliparas (Robson 1), one induced nullipara (Robson 2A) and one induced multipara (Robson 4A), and both spontaneous nullipara neonates were examined by the NICU team for more than 5 minutes. The other two were examined by the labor and delivery nurses and returned to SSC after more than 5 minutes apart.

### Uninterrupted SSC

All 48 (57.1%) of the remaining dyads had uninterrupted SSC, not interrupted by either staff or family.

### Widström's 9 stages of newborn behaviors

Of the 48 dyads who received immediate and uninterrupted SSC, 23 (47.9%) achieved the standard of best practice by demonstrating the instinctive behaviors described in Widström's 9 Stages and suckling in the first hour (the eighth stage), and 25 (52.1%) did not. Of the 15 nulliparas with spontaneous labor (Robson 1), 7 (46.7%) of the dyads achieved the standard and 8 (53.3%) did not. Of the six nulliparas who were induced (Robson 2A), none achieved the standard. Of the 14 multiparas with spontaneous labor (Robson 3), 10 (71.4%) of the dyads achieved the standard and 4 (28.6%) did not. Of the nine multiparas who were induced (Robson 4A), five (55.6%) of the dyads achieved the standards and four (44.4%) did not. Four (40%) of the 10 VBAC mothers (Robson 5) reached the final stage of the algorithm (3 with spontaneous labor and 1 induced). Of these, only one of the spontaneous labor dyads (5A) achieved the standard of suckling in the first hour after birth. Two spontaneous labor dyads and the one remaining induced dyad (5B) did not meet the standard.

Thirty-six (42.9%) dyads of the total number of participants in this study did not get either immediate or uninterrupted SSC after vaginal birth. Ten (11.9%) of these dyads required emergent cesarean section. Eleven (13.1%) required emergent care before beginning SSC and 10 (11.9%) received delayed SSC (these comprised the 31 dyads classified as “Emergent Care for Mother or Infant”). Five (6%) dyads were removed for care (emergent or routine) for more than 5 minutes during the hour (three were eventually returned to the mother).

The study protocol dictated that filming be continued if less than 8 minutes of separation occurred between mother and infant. The Algorithm specified recategorization based on a separation of more than 5 minutes. As a result, there are a number of infants included in the analysis who were separated for more than 5 but less than 10 minutes.

Ten newborns (11.9%) experienced a delay of more than 5 minutes before being placed skin to skin with their mother, but were reunited within 10 minutes after birth. Of those who were removed from the Algorithm pathway, only three eventually suckled (30%). Of the 12 infants who were delayed less than 5 minutes, 4 (33%) did suckle within the first hour. Of the five infants who did not have continuous SSC, the one removed for routine care did not suckle. Of the four removed for emergent concerns, one was later removed by NICU for more than 10 minutes (so filming stopped) and three were returned within 10 minutes (and filming continued). Of those three, one suckled within the hour and two did not.

Neonates who were born after labor inductions were the least likely to follow the stages to achieve breastfeeding in the first hour after birth. Thirty-five (41.7%) of the 84 participants were inductions, 16 nulliparas, and 19 multiparas. Of these neonates, only 5 (14.7%) reached the suckling stage (Widström's eighth stage). Of the 50 (60%) mothers who began labor spontaneously (23 nulliparous and 27 multiparous), 18 (36%) reached the suckling stage.

Review of the four dyads with para unknown reveals that two did not receive immediate SSC due to emergent care for mother or infant, one experienced immediate, continuous, uninterrupted SSC but did not suckle, and one experienced immediate, continuous, uninterrupted SSC and did suckle. They are included in Table 3 as “para unrecorded” but not in the Algorithm.

## Discussion

Lack of initiation and lack of consistency are barriers to the universal application of SSC. Improvement in the performance of a healthcare practice “requires measures that reflect the richness and complexity of the phenomenon under scrutiny”^29^ (p.2) beginning with generalizable scientific evidence, the selection of a particular context, and a realistic and efficient measurement of performance. In the case of SSC immediately after birth and early breastfeeding, the generalizable scientific evidence indicates that SSC improves maternal birth outcomes as well as decreases infant morbidity and mortality. The context for this study was a Baby-Friendly designated hospital with a standard of care that included immediate, uninterrupted SSC for all stable babies and mothers after both vaginal and cesarean births and staff education provided to support this standard. In this study, the performance measurement of SSC (immediate, continuous, uninterrupted) utilized a process algorithm (HCP-S2S-IA), which included Robson criteria, acknowledging the richness and complexity of the system being analyzed. The process allows for detection of specific limitations in the practice, illuminating barriers to the achievement of the standard of practice and highlighting opportunities to support the standard.

Although mothers could only be consented into the study if they were expected to give birth vaginally and had no medical concerns that would preclude eligibility for SSC in the first hour after birth, 31 of 84 newborns (36.9%) either did not achieve a vaginal birth due to emergent cesarean or were unable to receive SSC after a vaginal birth. The Algorithm highlights these numbers and provides an opportunity for further exploration. Why are so many mothers and babies that were categorized as “healthy” during labor unable to experience SSC immediately after the birth? Five neonates were removed during the first hour after birth. The Algorithm highlights an opportunity for additional training of staff. Could these examination and resuscitation procedures that took less than 5 minutes have taken place while the baby remained in SSC with mom? Only 23 (27.4%) of the 84 newborns self-attached and suckled after vaginal birth. The process map illuminated patterns for closer scrutiny, including a large percentage of neonates and mothers who entered labor with no medical concerns but who required emergent care, many of whom had delayed or no SSC.

Another pattern noted is the divergent pathway of dyads who had their labor induced. When viewed through the lens of this Algorithm, induction clearly emerges as a negative influence in achieving the optimal goal of suckling in the first hour after birth. Of the 34 inductions, 21 (61.8%) were separated immediately after birth, while 22 (44%) of the noninductions were separated. Of newborns who reached the suckling stage, only 31.3% of the inductions (Robson criteria 2A, 4A, and 5B) did suckle within the first hour after birth, whereas 68.8% did not suckle. With the spontaneous labor dyads, 56.6% did suckle and 43.8% did not suckle within the first hour after birth.

### Limitations

The study is limited by a single location and by sample size, which could limit generalizability. Analysis is limited by the use of only Robson criteria and the clinical pathway as correlation parameters. Augmentation of labor with synthetic oxytocin is not reflected in this analysis, since it is not an element of Robson criteria, nor is epidural medication. We have presented data in another analysis of this study^30^ showing that as the dose of fentanyl and synthetic oxytocin increased, the probability of reaching the eighth stage (suckling) of Widström's 9 Stages in the first hour after birth while in SSC with mother decreased.

## Conclusions

Immediate, continuous, and uninterrupted SSC has been shown to support newborns in reaching the goal of suckling in the first hour after birth, while induction of labor has been shown to reduce rates of suckling in the first hour. The Algorithm presented provides a tool to help determine areas where specific interventions would be most effective, that is, reducing delays or decreasing interruptions during SSC.

Not suckling in the first hour after birth places newborns at higher risk for neonatal morbidities and mortality. Examining patterns and developing strategies for change optimizes patient outcomes. Making considered and iterative changes that will lead to better patient outcomes requires accurate and powerful measurement of what is actually happening, how work is conducted in the usual way, and pattern analysis as described in this article. This process can lead to opportunities for improvement with targeted interventions. The next steps are review, reflection, and the development of specific plans for change.

## References

[1] World Health Organization. Implementation Guidance: Protecting, Promoting and Supporting Breastfeeding in Facilities Providing Maternity and Newborn Services—The Revised Baby-Friendly Hospital Initiative. Geneva: World Health Organization, 2018 Report No.: CC BY-NC-SA 3.0 IGO

[2] Baby-Friendly USA. Guidelines and Evaluation Criteria for Facilities Seeking Baby-Friendly Designation. Baby-Friendly USA. 2016 Available at: www.babyfriendlyusa.org/get-started/the-guidelines-evaluation-criteria (accessed 718, 2018)

[3] Moore ER, Bergman N, Anderson GC, et al. Early skin-to-skin contact for mothers and their healthy newborn infants. Cochrane Database Syst Rev 2016;25;11:CD003519 10.1002/14651858.CD003519.pub4 PMC6464366 27885658

[4] Bystrova K, Widström AM, Matthiesen AS, et al. Skin-to-skin contact may reduce negative consequences of “the stress of being born”: A study on temperature in newborn infants, subjected to different ward routines in St. Petersburg. Acta Paediatr 2003;92:320–326 12725547 10.1080/08035250310009248

[5] Takahashi Y, Tamakoshi K, Matsushima M, et al. Comparison of salivary cortisol, heart rate, and oxygen saturation between early skin-to-skin contact with different initiation and duration times in healthy, full-term infants. Early Hum Dev 2011;87:151–157 21220191 10.1016/j.earlhumdev.2010.11.012

[6] Beiranvand S, Valizadeh F, Hosseinabadi R, et al. The effects of skin-to-skin contact on temperature and breastfeeding successfulness in full-term newborns after cesarean delivery. Int J Pediatr 2014;2014:1–7 10.1155/2014/846486 PMC4291124 25610472

[7] Christensson K, Cabrera T, Christensson E, et al. Separation distress call in the human neonate in the absence of maternal body contact. Acta Paediatr 1995;84:468–473 7633137 10.1111/j.1651-2227.1995.tb13676.x

[8] Mazurek T, Mikiel-Kostyra K, Mazur J, et al. [Influence of immediate newborn care on infant adaptation to the environment]. Med Wieku Rozwoj 1999;3:215–224 10910653

[9] Marín Gabriel MÁ, del Rey Hurtado de Mendoza B, Jiménez Figueroa L, et al. Analgesia with breastfeeding in addition to skin-to-skin contact during heel prick. Arch Dis Child Fetal Neonatal Ed 2013;98:F499–F503 23839984 10.1136/archdischild-2012-302921

[10] Essa RM, Abdel Aziz Ismail NI Effect of early maternal/newborn skin-to-skin contact after birth on the duration of third stage of labor and initiation of breastfeeding. J Nurs Educ Pract 2015;5 Available at www.sciedu.ca/journal/index.php/jnep/article/view/5698 (accessed 58, 2017)

[11] Dordević G, Jovanović B, Dordević M [An early contact with the baby—Benefit for the mother]. Med Pregl 2008;61:576–579 19368275 10.2298/mpns0812576d

[12] Aghdas K, Talat K, Sepideh B Effect of immediate and continuous mother-infant skin-to-skin contact on breastfeeding self-efficacy of primiparous women: A randomised control trial. Women Birth 2014;27:37–40 24216342 10.1016/j.wombi.2013.09.004

[13] Righard L, Alade MO Sucking technique and its effect on success of breastfeeding. Birth 1992;19:185–189 1472265 10.1111/j.1523-536x.1992.tb00399.x

[14] Bramson L, Lee JW, Moore E, et al. Effect of early skin-to-skin mother—Infant contact during the first 3 hours following birth on exclusive breastfeeding during the maternity hospital stay. J Hum Lact 2010;26:130–137 20110561 10.1177/0890334409355779

[15] Crenshaw JT, Cadwell K, Brimdyr K, et al. Use of a video-ethnographic intervention (PRECESS immersion method) to improve skin-to-skin care and breastfeeding rates. Breastfeed Med 2012;7:69–78 22313390 10.1089/bfm.2011.0040

[16] Mahmood I, Jamal M, Khan N Effect of mother-infant early skin-to-skin contact on breastfeeding status: A randomized controlled trial. J Coll Physicians Surg Pak 2011;21:601–605 22015120 10.2011/JCPSP.601605

[17] Marín Gabriel MA, Llana Martín I, López Escobar A, et al. Randomized controlled trial of early skin-to-skin contact: Effects on the mother and the newborn. Acta Paediatr 2010;99:1630–1634 19912138 10.1111/j.1651-2227.2009.01597.x

[18] Mikiel-Kostyra K, Mazur J, Bołtruszko I Effect of early skin-to-skin contact after delivery on duration of breastfeeding: A prospective cohort study. Acta Paediatr 2002;91:1301–1306 12578285 10.1080/08035250216102

[19] Widström A-M, Lilja G, Aaltomaa-Michalias P, et al. Newborn behaviour to locate the breast when skin-to-skin: A possible method for enabling early self-regulation. Acta Paediatr 2011;100:79–85 20712833 10.1111/j.1651-2227.2010.01983.x

[20] Smith ER, Hurt L, Chowdhury R, et al. Delayed breastfeeding initiation and infant survival: A systematic review and meta-analysis. PLoS One 2017;12:e0180722 28746353 10.1371/journal.pone.0180722 PMC5528898

[21] UNICEF. From the first hour of life: Making the case for improved infant and young child feeding everywhere. New York, NY: UNICEF, 2016 Available at https://data.unicef.org/wp-content/uploads/2016/10/From-the-first-hour-of-life.pdf (accessed 718, 2018)

[22] De Chateau P, Wiberg B Long-term effect on mother-infant behaviour of extra contact during the first hour post partum. II. A follow-up at three months. Acta Paediatr Scand 1977;66:145–151 842336 10.1111/j.1651-2227.1977.tb07826.x

[23] Vaidya K, Sharma A, Dhungel S Effect of early mother-baby close contact over the duration of exclusive breastfeeding. Nepal Med Coll J 2005;7:138–140 16519083

[24] Centers for Disease Control & Prevention. Breastfeeding Report Card: 2016 Centers for Disease Control & Prevention, Division of Nutrition, Physical Activity, and Obesity, National Center for Chronic Disease Prevention and Health Promotion. 2016 Available at https://www.cdc.gov/breastfeeding/pdf/2016breastfeedingreportcard.pdf (accessed 718, 2018)

[25] Robson M. Classification of caesarean sections. Fetal Matern Med Rev 2001;12 Available at: www.journals.cambridge.org/abstract_S0965539501000122 (accessed 51, 2018)

[26] Brimdyr K, Cadwell K, Stevens J, et al. An implementation algorithm to improve skin-to-skin practice in the first hour after birth. Matern Child Nutr 2017;e12571 29230957 10.1111/mcn.12571 PMC5900969

[27] Brimdyr K, Wiström AM, Svensson K Skin to Skin in the First Hour after Birth: Practical Advice for Staff After Vaginal and Cesarean Birth [DVD]. Sandwich, MA: Healthy Children Project, Inc., 2011

[28] Farine D, Shepherd D Classification of caesarean sections in Canada: the modified Robson criteria. J Obstet Gynaecol Can 2012;34:976–979 23067954 10.1016/S1701-2163(16)35412-3

[29] Batalden PB, Davidoff F What is “quality improvement” and how can it transform healthcare? Qual Saf Health Care 2007;16:2–3 17301192 10.1136/qshc.2006.022046 PMC2464920

[30] Brimdyr K, Cadwell K, Widström A-M, et al. The association between common labor drugs and suckling when skin-to-skin during the first hour after birth. Birth 2015;42:319–328 26463582 10.1111/birt.12186 PMC5057303

